# Seven-year mortality in heart failure patients with undiagnosed diabetes: an observational study

**DOI:** 10.1186/1475-2840-10-39

**Published:** 2011-05-14

**Authors:** Juana A Flores-Le Roux, Josep Comin, Juan Pedro-Botet, David Benaiges, Jaume Puig-de Dou, Juan J Chillarón, Alberto Goday, Jordi Bruguera, Juan F Cano-Perez

**Affiliations:** 1 Department of Endocrinology, Hospital del Mar, Departament de Medicina, Universitat Autónoma de Barcelona, Spain; 2 Department of Endocrinology, Hospital del Mar, Paseo Marítimo, 25-29 E-08003 Barcelona, Spain

**Keywords:** acute heart failure, diabetes, cardiovascular mortality, undiagnosed diabetes

## Abstract

**Background:**

Patients with type 2 diabetes mellitus and heart failure have adverse clinical outcomes, but the characteristics and prognosis of those with undiagnosed diabetes in this setting has not been established.

**Methods:**

In total, 400 patients admitted consecutively with acute heart failure were grouped in three glycaemic categories: no diabetes, clinical diabetes (previously reported or with hypoglycaemic treatment) and undiagnosed diabetes. The latter was defined by the presence of at least two measurements of fasting plasma glycaemia ≥ 7 mmol/L before or after the acute episode.Group differences were tested by proportional hazards models in all-cause and cardiovascular mortality during a 7-year follow-up.

**Results:**

There were 188 (47%) patients without diabetes, 149 (37%) with clinical diabetes and 63 (16%) with undiagnosed diabetes. Patients with undiagnosed diabetes had a lower prevalence of hypertension, dyslipidaemia, peripheral vascular disease and previous myocardial infarction than those with clinical diabetes and similar to that of those without diabetes. The adjusted hazards ratios for 7-year total and cardiovascular mortality compared with the group of subjects without diabetes were 1.69 (95% CI: 1.17-2.46) and 2.45 (95% CI: 1.58-3.81) for those with undiagnosed diabetes, and 1.48 (95% CI: 1.10-1.99) and 2.01 (95% CI: 1.40-2.89) for those with clinical diabetes.

**Conclusions:**

Undiagnosed diabetes is common in patients requiring hospitalization for acute heart failure. Patients with undiagnosed diabetes, despite having a lower cardiovascular risk profile than those with clinical diabetes, show a similar increased mortality.

## Introduction

Type 2 diabetes has an estimated prevalence of 20-40% in heart failure patients, being an independent risk factor not only for the development of heart failure [1-6] but also for increased morbidity and mortality [7-13].

On the other hand, several studies have highlighted the frequent underdiagnosis of diabetes in the general population and in high cardiovascular risk patients [14-17]. The few reports on the prevalence of undiagnosed diabetes in patients with stable chronic heart failure suggest it could affect 10% of patients [18,19].

Most of the studies that have quantified the adverse outcomes of patients with diabetes and heart failure have been limited to patients with a known diagnosis of diabetes. Previous reports have outlined the prognostic importance of undiagnosed diabetes in patients with different cardiovascular diseases [16,20-22]. In patients with established coronary artery disease, undiagnosed diabetes has been proven to be a highly significant and independent predictor of cardiovascular and all-cause mortality [21,22]. Patients with heart failure requiring hospital admission for an acute episode represent a high-risk population for adverse outcomes [23-26]. In this group of patients, mortality is higher than in those with chronic stable heart failure and, thus, identification of patients with an increased risk within this group who could benefit from more aggressive therapeutic interventions could help improve their poor outcomes. However, the risk associated with undiagnosed diabetes in patients with acute heart failure has not been described. Thus, the aim of the present study on patients hospitalized for acute heart failure was to determine the prevalence and characteristics of patients with undiagnosed diabetes and its impact on all-cause and cardiovascular mortality during a 7-year follow-up in comparison with patients with and without clinical diabetes.

## Patients and Methods

### Patients and baseline measurements

An observational study of a retrospective cohort of all patients admitted to the cardiology department of Hospital del Mar (Barcelona, Spain) with the diagnosis of acute heart failure was carried out between January 1st, 2000 to December 31st, 2002. All patients with acute heart failure as one of the two first discharge diagnoses were included. Discharge records were reviewed and the following data was gathered: demographic and clinical characteristics, cardiovascular risk factors, previous diabetes treatment, cause of heart failure, left ventricular ejection fraction measured by echocardiography, chronic renal failure, peripheral vascular disease, previous myocardial infarction or stroke, plasma biochemical parameters at the time of admission [glycemia, creatinine, hemoglobin and glycosylated hemoglobin (HbA_1c_)] and drug therapy at discharge. Before the year 2002 HbA_1c _was only determined in patients with a clinical diagnosis of diabetes at admission. From January 2002 onwards, HbA_1c _was systematically measured in all subjects admitted with acute heart failure. Thus, data on HbA_1c _for individuals without clinical diabetes was available in only 35% of the cases. Investigators obtained data from medical and laboratory records and did not participate in patient treatment and management.

To identify undiagnosed diabetes, we had access to clinical diagnosis, laboratory data and pharmaceutical treatment registered in all primary health care centres in the province of Barcelona and in the autonomous community of Catalonia. Laboratory data for blood samples drawn in acute situations are specified as "emergency laboratory", as these blood samples are processed in a different laboratory, and thus these samples were excluded for diabetes diagnosis. For blood samples drawn in primary health care centres at routine check-up visits, patients are given specific instructions regarding fasting a minimum of 8 hours, as per protocol. In patients presenting hyperglycaemia during admission, but no prior glucose values in the range of diabetes, we also reviewed the laboratory data of the year after discharge to rule out recent-onset diabetes.

Diabetes was diagnosed according to 1997 American Diabetes Association criteria [27], and patients were classified in three categories: 1) clinical diabetes mellitus, when the diagnosis was specified in medical reports or patients were being treated for diabetes (dietary advice, oral drugs or insulin); 2) undiagnosed diabetes mellitus, without clinical diabetes but with two or more outpatient fasting plasma glucose concentrations ≥ 7 mmol/L; and 3) no diabetes mellitus, who did not meet the criteria for clinical or undiagnosed diabetes.

### Mortality data

Mortality rates and causes of death were monitored every year until January 2009 by systematic review of hospital records and death certificates from the Death Registry Office of Catalonia, coded according to the International Classification of Diseases (ICD) 9th edition. Cardiovascular death was defined by ICD-9 codes 390-459. Mean follow-up was 7.5 year (range: 6-9 years). The study was approved by the local Ethics Committee.

### Statistical analysis

Quantitative variables were described by mean ± standard deviation (SD) and compared by Student's two-tailed T test. Qualitative variables were described by frequencies and percentages and were compared using Chi-Square test or Fisher's exact test, as appropriate. Logistic regression model was performed to assess specific characteristics of patients with undiagnosed diabetes. Survival univariate analysis was estimated with the Kaplan-Meier method and differences were tested with a log rank test. Multivariate survival analysis was performed with Cox proportional hazards model, to determine the independent contributions of the three glycaemic categories to all-cause and cardiovascular mortality after adjusting for age, sex, comorbidities (smoking, hypertension, dyslipidaemia, stroke, chronic renal failure, peripheral vascular disease, previous myocardial infarction) hemoglobin concentrations, left ventricular ejection fraction and standard heart failure medication (loop diuretic/thiazide, beta-blocker, angiotensin converting enzyme-inhibitor/angiotensin receptor blocker). Values of p < 0.05 were considered significant. Statistical analysis was conducted using SPSS 15.0 (SPSS, Inc., Chicago, Illinois).

## Results

### Baseline characteristics

416 patients were initially included and 16 cases (3.9%) were excluded from analyses for being lost to follow-up. The final study group included 400 patients, 203 men and 197 women, with a mean age of 71.5 ± 10 years. The distribution of patients within glycaemic categories was: clinical diabetes 37%, undiagnosed diabetes 16% and no diabetes 47%. Regarding diabetes therapy previous to admission in patients with clinical diabetes, 30% received dietary advice only, 48% took oral antidiabetic agents and 30% used insulin. Baseline characteristics of patients are shown in Table 1. Clinical diabetes was associated with a greater prevalence of hypertension, dyslipidaemia, peripheral vascular disease and previous myocardial infarction. Patients with undiagnosed diabetes had a prevalence of these comorbidities similar to that of those without diabetes. Patients with diabetes, both clinical and undiagnosed, had more hospital admissions due to acute heart failure in the previous year; however, no differences in left ventricular ejection fraction were found among groups. Furthermore, the frequency of heart failure with systolic dysfunction (ejection fraction less than 40%) was also similar among groups (51.5% in no diabetes, 45.8% in undiagnosed diabetes and 52.3%, in clinical diabetes, p = 0.67). Regarding laboratory parameters at admission, hemoglobin concentrations were significantly lower in patients with diabetes, both clinical and undiagnosed, compared to those without diabetes. Fasting glycaemia was higher in subjects with clinical diabetes than in those with undiagnosed diabetes. HbA_1c _was 62 ± 16 mmol/mol (7.8 ± 1.5%) in patients with clinical diabetes, 44 ± 12 mmol/mol (6.2 ± 1.1%) in subjects with undiagnosed diabetes and 40 ± 16 mmol/mol (5.8 ± 1.5%) in those without diabetes (p < 0.001 among groups). Cardiovascular drug therapy at discharge was similar in the three groups, although beta-blockers were more commonly prescribed in patients with clinical diabetes.

**Table 1:** Baseline characteristics of patients according to glycemic status at hospitalization

	No diabetes (n = 188)	Undiagnosed diabetes (n = 63)	Clinical diabetes (n = 149)	p-value
Age (years)	71.1 (11.4)	72.7 (10.4)	71.4 (7.7)	0.568

Male (%)	100 (53)	30 (49)	73 (48)	0.627

Cardiovascular risk factors				
Smoking (%)	37 (19.8)	7 (11.3)	19 (12.6)	0.113
Hypertension (%)	129 (69)	44 (71)	126 (83.4)	0.007
Dyslipidemia (%)	40 (21.3)	17 (27)	56 (37.6)	0.002

Previous cardiovascular disease				
MI (%)	39 (21)	18 (29)	51 (34)	0.024
Stroke (%)	20 (11)	7 (11)	23 (15)	0.434
Peripheral vascular disease (%)	16 (9)	4 (6)	31 (21)	0.001

HF ischemic etiology (%)	65 (35)	25 (40)	80 (54)	0.002

LVEF % (SD)	46 ± 18	47 ± 18	45 ± 18	0.771

Last-year HF Admission (%)	18 (9.6)	14 (22.6)	31 (20.7)	0.006

Biochemical parameters at admission				
Fasting glucose mmol/L (SD)	5.5 ± 0.8	6.6 ± 1.5	7.8 ± 2.7	< 0.001
Creatinine μmol/L (SD)	116.7 ± 73.4	120.2 ± 49.5	114 ± 55.7	0.817
Haemoglobin g/L (SD)	0.131 ± 0.020	0.124 ± 0.023	0.125 ± 0.019	0.015

HF drug therapy at discharge				
ACEI/ARA II (%)	122 (65.2)	39 (62.9)	97 (64.2)	0.943
β-blockers (%)	52 (27.8)	13 (21)	56 (37.1)	0.041
Diuretics (%)	163 (87.2)	52 (83.9)	126 (83.4)	0.597
Spirinolactone (%)	55 (29.4)	23 (37.1)	47 (31.1)	0.527

ACEI, angiotensin-converting enzyme inhibitors; ARA-II, angiotensin-2 receptor antagonists; HF, heart failure; LVEF, left ventricular ejection fraction; MI, myocardial infarction.

When comparing subjects with undiagnosed diabetes with those without diabetes in multivariate logistic regression analysis, only hospital admission for heart failure in the previous year was more frequent in those with undiagnosed diabetes [risk ratio of 1.37 (95% confidence interval (CI): 1.16 to 1.49)].

### Mortality during follow-up

**Figure 1:**
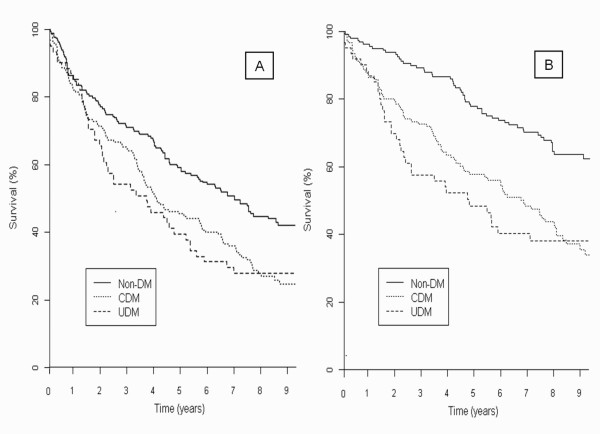
Kaplan-Meier survival curves for all cause (A) and cardiovascular (B) mortality* according to glycaemic category (no-diabetes, clinical diabetes and undiagnosed diabetes). No-DM: no-diabetes mellitus; CDM: clinical diabetes mellitus; UDM: undiagnosed diabetes mellitus. *Data are adjusted for age, sex, comorbidities, haemoglobin concentrations, left ventricular ejection fraction and standard heart failure medication.

The 7-year incidence of total and cardiovascular mortality in relation to baseline glycaemic categories is shown in Table 2. There were 262 (65.2%) deaths, of which 192 were cardiovascular. The Kaplan-Meier survival curves illustrate higher risk for all-cause and cardiovascular death in patients with clinical and undiagnosed diabetes (Figure 1).

**Table 2:** Adjusted hazards ratio (HR) for all-cause and cardiovascular death at 7-year follow-up in heart failure patients with undiagnosed and clinical diabetes compared with those without diabetes

	All-cause death		p value	Cardio-vascular death		p value
	No. of events (%) HR*(95% CI)			No. of events (%) HR*(95% CI)		
No-diabetes (n = 188)	105 (55.9)	1 (reference)		56 (29.8)	1 (reference)	

Undiagnosed diabetes (n = 63)	45 (72.6)	1.69 (1.17-2.46)	0,006	36 (58.1)	2.45 (1.58-3.81)	0,001

Clinical diabetes (whole group)	112 (74.2)	1.48 (1.10-1.99)	0,009	84 (55.6)	2.01 (1.40-2.89)	0,006

Clinical diabetes (treatment)						

Diet alone (n = 43)	28 (63.6)	1.38 (0.9-2.13)	0,129	20 (45.5)	1.87 (1.09-3.18)	0,026

Oral drugs (n = 48)	34 (70.8)	1.33 (0.89-1.98)	0,221	22 (45.8)	1.49 (0.89-2.47)	0,096

Insulin (n = 58)	50 (84.7)	2.11 (1.48-3.00)	0,005	42 (71.2)	2.73 (1.76-4.24)	< 0,001

*Adjusted for age, sex, hypertension, dyslipidemia, smoking, previous stroke, previous myocardial infarction, peripheral vascular disease, hemoglobin and plasma creatinine concentrations.

**Figure 2:**
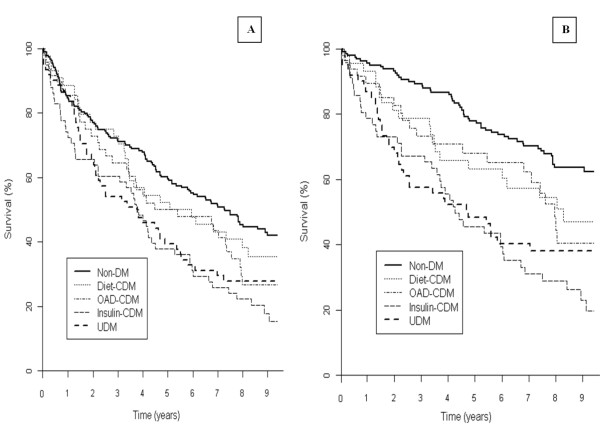
Kaplan-Meier survival curves for all cause (A) and cardiovascular (B) mortality* according to glycaemic status and diabetes treatment (no-diabetes, undiagnosed diabetes, clinical diabetes on diet, clinical diabetes on oral drugs and clinical diabetes on insulin). No-DM: no-diabetes mellitus; CDM: clinical diabetes mellitus; UDM: undiagnosed diabetes mellitus. *Data are adjusted for age, sex, comorbidities, haemoglobin concentrations, left ventricular ejection fraction and standard heart failure medication.

Cox proportional hazards models comparing patients with undiagnosed and clinical diabetes with those without diabetes are depicted in Table 2. All-cause mortality risk was similar in subjects with undiagnosed and clinical diabetes and significantly higher than in those without diabetes, with an adjusted risk ratio of 1.69 (95% CI: 1.17-2.46) and 1.48 (95% CI: 1.10-1.99), respectively. Patients with undiagnosed and clinical diabetes also showed an increased risk for cardiovascular death, with an adjusted risk ratio of 2.45 (95% CI: 1.58-3.81) and 2.01 (95% CI: 1.40-2.89), respectively. When patients with clinical diabetes were stratified by antidiabetic therapy, only those on insulin treatment showed significantly increased all-cause mortality (figure 2), with an adjusted risk ratio of 2.11 (95% CI: 1.48-3.00). This subgroup of patients also had the highest risk of death from a cardiovascular cause when compared with subjects without diabetes [adjusted risk ratio 2.73 (95% CI: 1.76-4.24)], table 2.

## Discussion

A high proportion of subjects with diabetes were previously undiagnosed, consequently, the prevalence of diabetes in patients with acute heart failure is higher than previously recognized, occurring in more than half of the patients studied. Furthermore, the study established that patients with undiagnosed diabetes, despite having less cardiovascular risk factors and comorbidities, had a similar increased mortality as that of subjects with clinical diabetes.

### Prevalence of undiagnosed diabetes

The prevalence of diabetes in this study was higher than previously reported, which ranged from 23% to 43% [10-13]. This wide range reflects the diverse criteria used to identify patients with diabetes and the differences between heart failure populations analyzed. Most studies and registries of heart failure have only included patients with clinical diabetes, based on medical history or antidiabetic drug use [10-13]. This may exclude up to 20% of the population with diabetes following dietary advice only, as well as those with undiagnosed diabetes. In the present study, systematic "review" of fasting blood glucose levels resulted in an undiagnosed diabetes prevalence of 16%, which means that 30% of diabetes cases were not detected. This figure is consistent with the undiagnosed diabetes prevalence reported in subjects with established cardiovascular disease [16,17,21,22]. In patients with stable chronic heart failure, Kistorp et al [18] described a clinical diabetes prevalence of 21%, while that of undiagnosed diabetes was 6%. Furthermore, most diabetes prevalence studies in heart failure have been conducted in outpatient clinics, while the present study only included those requiring hospitalization for acute decompensated heart failure. In the large US ADHERE registry of patients with acute decompensated heart failure, 40% of the patients had clinical diabetes [13], similar to the 37% prevalence reported in the present study. Therefore, the prevalence of diabetes in heart failure patients seems to be increased in those requiring hospitalization for acute decompensation.

### Long-term mortality of patients with undiagnosed diabetes

Patients with undiagnosed diabetes were 1.69 times (95% CI: 1.16-2.35) more likely to die during follow-up than those without diabetes. The excess in mortality appears to be mainly due to increased cardiovascular mortality. The present study demonstrates this relationship in a cohort of heart failure patients and the results are similar to those found in patients with coronary artery disease [21,22]. The association between undiagnosed diabetes and increased mortality existed despite the relative preservation of left ventricular ejection fraction. This finding is in agreement with Suskin et al [19], who found undiagnosed diabetes to be related to worse symptomatic status, but not worsening of left ventricular ejection fraction.

It is known that increased cardiovascular mortality in type 2 diabetes is related, at least in part, with comorbidities such as hypertension, dyslipidaemia and other cardiovascular diseases. However, in the present study, patients with undiagnosed diabetes had a mortality risk similar to those with clinical diabetes despite having lower fasting blood glucose and HbA_1c _levels at admission and a lower prevalence of hypertension, dyslipidaemia, coronary heart disease and peripheral vascular disease. Thus, the increased mortality in subjects with undiagnosed diabetes must be attributed to factors other than those usually associated with diabetes.

In this respect, previous studies showed heart failure to be an insulin-resistant state that may predispose to diabetes [28,29]. Amato et al [30] demonstrated in a longitudinal study that chronic heart failure was associated with an increased incidence of non-insulin-dependent diabetes mellitus. Moreover, previous evidence supports the idea that insulin resistance progresses within the natural course of heart failure [31]. Based on these findings, undiagnosed diabetes identified in the present study could reflect a "hyperglycemia" developed in patients with more severe heart failure, as reflected by its association with more previous hospitalizations for acute heart failure. Therefore, rather than being the cause of poor clinical outcomes, undiagnosed diabetes could represent a prognostic marker of heart failure severity.

To our knowledge, this is the first study to describe the prevalence of undiagnosed diabetes in patients with acute heart failure and to analyze the impact of this condition on long-term mortality. The clinical relevance of our findings is that more attention should be paid in diagnosing glucose abnormalities in patients admitted with heart failure, as this simple, cost-effective intervention allows identification of high-risk patients who could benefit from more aggressive therapeutic interventions.

Our study has some limitations that need to be acknowledged. The retrospective collection of clinical data precluded gathering information that might have been used to more accurately assess the relationship between diabetes and mortality in patients with heart failure, such as diabetes duration, complete data on HbA1c and microangiopathic complications. However, the retrospective collection of clinical data allowed evaluation of the impact of undiagnosed diabetes; a prospective study would have prevented this analysis on ethical grounds. Furthermore, the cohort of the present study was restricted to patients with acute decompensated heart failure that required hospitalization, and thus the data observed here cannot be extrapolated to the whole heart failure population. Finally, we do not have information regarding the possible diagnosis and treatment of diabetes during the seven-year follow-up period in those patients with undiagnosed diabetes at the time of admission, and thus can not analyze potential differences. Therefore, further studies will be needed to ascertain whether early diagnosis and treatment of diabetes can improve clinical outcomes in these patients.

## Conclusions

Undiagnosed diabetes is common in patients requiring hospitalization for acute heart failure. Patients with undiagnosed diabetes, despite having a lower cardiovascular risk profile than those with clinical diabetes, show a similar increased mortality. These findings underscore the importance of identifying glucose abnormalities in heart failure patients. This simple and cost-effective intervention permits identification of high-risk patients who could benefit from aggressive therapeutic interventions.

## Consent

Written informed consent was obtained from the patient for publication of this study. A copy of the written consent is available for review by the Editor-in-Chief of this journal.

## List of Abbreviations

ACE: Angiotensin converting enzyme; CI: confidence interval; HbA_1c_: glycosylated hemoglobin; ICD: International classification of diseases; SD: standard deviation.

## Competing interests

The authors declare that they have no competing interests.

## Authors' contributions

JAFL and JC carried out the design of the study, participated in patient recruitment and elaborated the draft of the manuscript. DB participated in patient recruitment and in the design of the study. JJC performed the statistical analysis; JPD carried out patient recruitment and participated in the draft of the manuscript. AG and JP-B participated in the design of the study and coordination. JFC conceived the study and participated in design and coordination. All authors read and approved the final manuscript.
